# Spatiotemporal control of pattern formation during somitogenesis

**DOI:** 10.1126/sciadv.adk8937

**Published:** 2024-01-26

**Authors:** Cassandra McDaniel, M. Fethullah Simsek, Angad Singh Chandel, Ertuğrul M. Özbudak

**Affiliations:** ^1^ Division of Developmental Biology, Cincinnati Children’s Hospital Medical Center, Cincinnati, OH 45229, USA.; ^2^ Systems Biology and Physiology Graduate Program, University of Cincinnati College of Medicine, Cincinnati, OH 45229, USA.; ^3^ Department of Pediatrics, University of Cincinnati College of Medicine, Cincinnati, OH 45229, USA.

## Abstract

Spatiotemporal patterns widely occur in biological, chemical, and physical systems. Particularly, embryonic development displays a diverse gamut of repetitive patterns established in many tissues and organs. Branching treelike structures in lungs, kidneys, livers, pancreases, and mammary glands as well as digits and bones in appendages, teeth, and palates are just a few examples. A fascinating instance of repetitive patterning is the sequential segmentation of the primary body axis, which is conserved in all vertebrates and many arthropods and annelids. In these species, the body axis elongates at the posterior end of the embryo containing an unsegmented tissue. Meanwhile, segments sequentially bud off from the anterior end of the unsegmented tissue, laying down an exquisite repetitive pattern and creating a segmented body plan. In vertebrates, the paraxial mesoderm is sequentially divided into somites. In this review, we will discuss the most prominent models, the most puzzling experimental data, and outstanding questions in vertebrate somite segmentation.

## Teaser

A comprehensive review explores models, data, and outstanding questions in the field of vertebrate somite segmentation.

## INTRODUCTION

Somites are segmental structures composed of epithelial cells that encapsulate a core of mesenchymal cells, and they are located as paired tissue blocks on both sides of the caudal neural tube and notochord. Cells located in somites later differentiate into the cells of the vertebral column, skeletal muscle, or dermis. Thus, the somites act as the blueprint for the segmental structure of the vertebral column and additionally influence patterning of associated vasculature and peripheral nerves (*1*, *2*).

The number of somites is conserved within each species but vary an order of magnitude among species (*3*). Species-specific somite counts remain unaltered under substantial alterations of the total number of cells, ploidy, and cell volumes. Under these perturbations, anteroposterior lengths of somites scale with the overall size of individual embryos (*4**–**6*). Several models are proposed to explain these important features of somite segmentation (i.e., sequential and periodic segmentation, reproducible segment numbers, and scaling of segment lengths).

While some molecular mechanisms seem to be conserved among species, others are not. We propose that the fundamental mechanisms governing somite segmentation are conserved among vertebrates while auxiliary mechanisms might be adapted in a species-specific manner. Explaining the conservation of pattern formation across thousands of vertebrate species without conservation of the causal mechanism would be difficult. A successful model for somite segmentation should build upon the conserved fundamental mechanisms (so that it will work in all species) but should also be flexible to incorporate auxiliary mechanisms (so that it will explain species-specific regulations).

### INITIAL MODELS OF SOMITE SEGMENTATION

In this section, we will discuss four phenomenological models preceding the discoveries of dynamic signaling pathways regulating somite segmentation. These four models are the original clock and wavefront model, the cell cycle (CC) model, the phase shift (PS) model, and the reaction-diffusion (RD) model.

### The clock and wavefront model

**Fig. 1.**
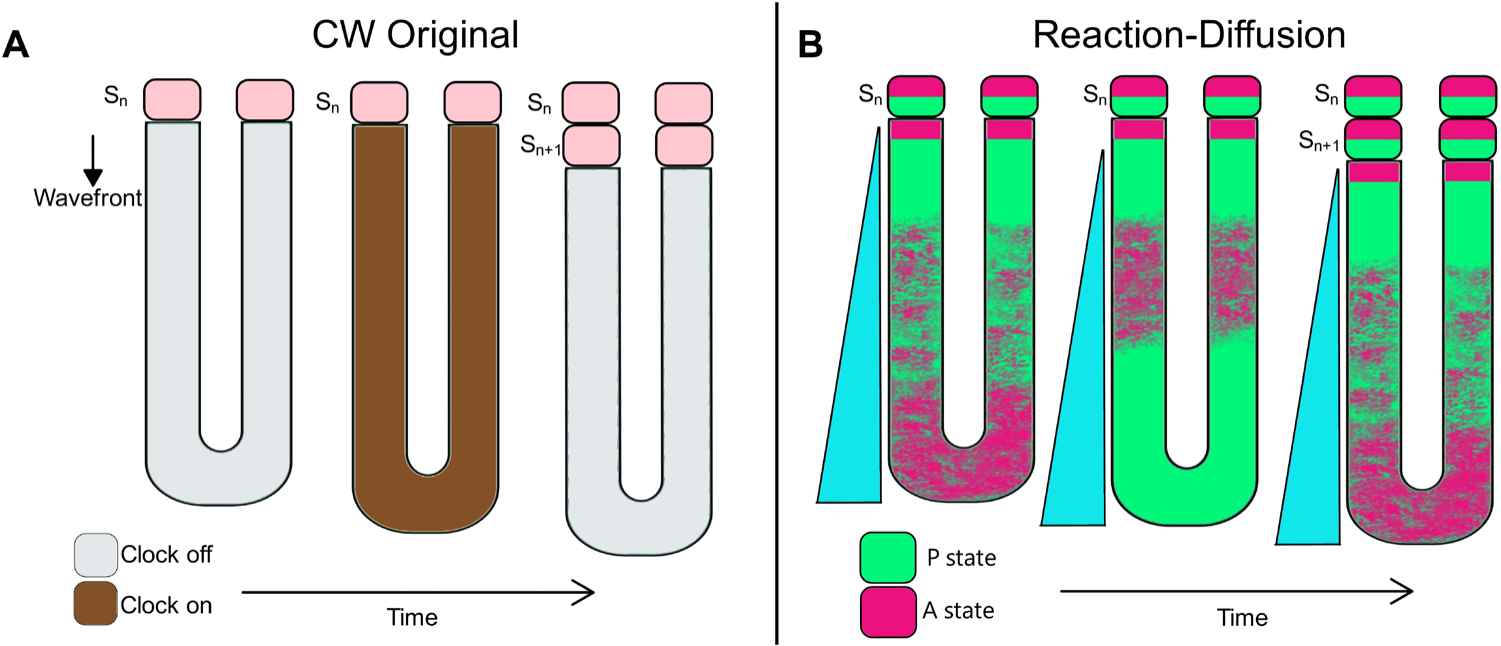
The original clock and wavefront model and the reaction-diffusion model. (**A**) The CW^O^ model: All cells are oscillating in synchrony, with the clock turning on and off in all cells in the PSM. Somites form at the anterior end of PSM (pink). An unknown maturation wavefront moves in anterior to posterior direction. (**B**) The RD model: Somites form because of the posterior oscillations between cell states P and A. When the level of a gradient (blue) drops below a certain threshold, oscillations are arrested and patterns form. This alternates between P and A states.

In 1976, Cooke and Zeeman proposed the clock and wavefront (CW^O^, “o” for original) model, which became the most influential and textbook model in the field. According to the CW^O^ model, the clock is a molecular oscillator synchronously oscillating in all presomitic mesoderm (PSM) cells (Fig. 1A). The wavefront is a front of cell-state change moving slowly in the antero-posterior direction. The clock somehow influences the probability of cell change and thereby rhythmically gating the slow progress of the wavefront. The CW^O^ model, conceptually, explains periodic and sequential segmentation of somites from the PSM. To explain segment length scaling, the speed of the wavefront is proposed to be proportional to the length of the embryo (*7*).

Several researchers applied single heat shock pulses to frog embryos to discover where the cells commit to segmentation in the PSM (*8**–**11*). They reasoned only uncommitted cells sensitive to the action of the wavefront will display segmentation defects upon heat shock–induced stress. After the beginning of a heat shock, several somites formed before the first defect in segmentation occurred. These results suggested that the hidden change of cellular state resulting in somite segmentation occurs several somite lengths posterior to the last segmented somite. These results were taken as indirect evidence supporting the CW^O^ model.

### The CC model

Stern and colleagues repeated the heat shock experiments in chicken embryos and observed segmentation defects like those originally observed in frog embryos. However, in approximately 15% of embryos, they also observed repetition of segmentation defects separated from each other by six to seven normal somites (*12*). Stern claimed that the periodic occurrence of segmentation defects argues against the idea that the heat shock reveals where the PSM cells respond to the wavefront and commit to form a somite (*2*). Stern and colleagues considered these low frequency of repeating segmentation defects as evidence of a clock. Because the duration of six to seven somite segmentation approximately corresponds to the CC in chicken embryos (9 to 10 hours), they proposed a CC-driven segmentation model in which there is no need for a wavefront. To explain the mismatch between the long CC period and short segmentation period, they hypothesized that cells only in a short window of CC are competent to induce somite segments (*13*). However, no molecular link has so far been established between these two periodic processes. Furthermore, a single heat shock in zebrafish also caused a low occurrence of periodic segmentation defects, but the periodicity of these defects did not match to the zebrafish CC (*14*). If the same heat shock perturbations cause similar periodic defects in both species, in our view, heat shock likely disrupts a conserved molecular process independent of the CC.

To explain the periodicity of defects, the CC model proposed that cells ingressing into the PSM are already in CC synchrony and they maintain their synchrony throughout the PSM. Stern and colleagues reported higher mitotic activity in the anterior PSM (aPSM) of chicken embryos and interpreted this as evidence of CC synchrony throughout PSM (*15*). However, CC synchrony has not been observed in human embryo models (*16*) and zebrafish (*17*), and mitotic cells are randomly distributed throughout the PSM in zebrafish (*18*). Moreover, the *emi1* zebrafish mutants, which prevent mitosis, showed that CC progression is not needed for initial segmentation of somites but is important for somite morphogenesis and maintenance of boundaries (*19*).

### The PS model

In 1969, Goodwin and Cohen proposed a general PS model to explain spatiotemporal pattern formation in many tissues, including somites (*20*). The PS model posits the existence of two clocks moving at different speeds from a pacemaker region (posterior PSM) toward the other end of the tissue. The faster propagating clock provides the temporal information. In contrast to the CW^O^ model, the PS model does not involve a wavefront but instead proposes that the positional information of boundary determination is encoded by the PS between the two clocks. Unexpectedly, the PS model has not received much attention in the somitogenesis field until recently (*21*, *22*). We will later compare the CW^O^ and PS models in greater detail.

### The RD model

Meinhardt proposed a RD model to explain somite segmentation (*23*, *24*). This model combines two diffusible molecules that autoactivate themselves and inhibit each other at short range, while reinforcing each other at long ranges. PSM cells thereby oscillate between two cellular states (A for anterior and P for posterior identity). This model also proposes that a posteroanterior morphogen gradient supports out of phase oscillations of A and P states. Cells located below a threshold of this gradient cannot maintain stable oscillations, and gradually slow down and arrest the oscillations. Thereby, cells settle down in stable A and P states in consecutive groups along the axis (Fig. 1B). The combination of one A and one P cell population corresponds to a single somite, while A and P compartments separately correspond to anterior and posterior halves of each somite. Both the CW^O^ and RD models contain a clock. But the clock in the RD model requires two diffusible molecules. In addition, the gradient in the RD model is acting hierarchically upstream of the clock, whereas the clock and the wavefront are independent in the CW^O^ model.

### DISCOVERIES OF THE SEGMENTATION CLOCK AND SIGNALING GRADIENTS

In this section, we will discuss the discovery of the segmentation clock and three signaling pathways establishing gradients along the PSM.

### Discovery of the first segmentation clock gene

**Fig. 2.**
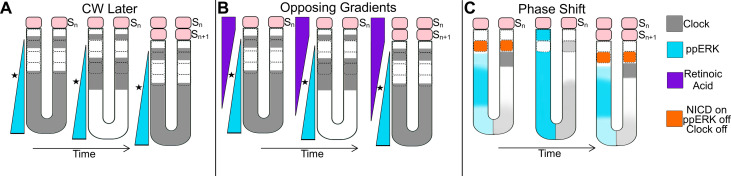
The later-updated clock and wavefront model, the opposing gradients clock and wavefront model, and the phase shift model. (**A**) The CW^L^ model: The clock oscillations display kinematic waves, and the wavefront is composed of a posterioanterior signaling gradient (e.g., FGF/ppERK). (**B**) The CW^OG^ model: The positional information is determined by the opposing activity of RA and FGF/ppERK (and Wnt). (**C**) The PS model: The striped expression of the clock encodes the spatial information, and the ppERK oscillations provide the temporal information. In mid-PSM, a group of cells are released from ppERK inhibition and receive Notch activation, where the future somite is determined (orange). The solid pink boxes are formed somites. The dashed boxes are predetermined somites.

Several other models are proposed preceding molecular discoveries; for a while, none of them established superiority over the other (*1*, *2*). In 1997, Pourquie and colleagues found the first clock gene, *c-hairy1*, with oscillatory mRNA expression in the PSM whose period of cyclical expression matched that of somite segmentation in chicken embryos (*25*). This landmark study showed that oscillations of *c-hairy1* mRNA expression are not synchronous among all PSM cells as the CW^O^ model posits but rather establish kinematic waves that sweep from the posterior to aPSM as the PS model posits (Fig. 2A). Yet the PS model was not cited, and the cyclic transcription of *c-hairy1* was viewed as providing molecular support for the CW^O^ model (*25*).

Pourquie and colleagues proposed that the observed pattern of *c-hairy1* expression can be explained by a kinematic clock-and-wavefront model, in which the wavefront smoothly slows down and lastly freezes the clock (*25*). Cooke wrote a perspective paper citing this landmark discovery and, therein, he noted the similarity between the coherently phase-shifted *c-hairy1* oscillations and the kinematic clocks in the PS model. Cooke also noted that the later adaptation of the CW model by Pourquie and colleagues (CW^L^, L for “later”) differs from what was originally proposed (CW^O^): “*A possible confusion arises because the phase shift of the clock revealed by chairy1 causes posterior-to-anterior wavefront appearances, whereas the wavefront of the original ‘clock and wavefront’ model is required to be from anterior to posterior and slow, advancing just once down the body during development… The original concept was of a less integrated sort than this, having ‘clock’ and ‘wavefront’ merely passing like ships in the night to leave the somite pattern, but having little else to do with each other during development.*” (*26*).

The clock and wavefront were independent in the CW^O^ model, while the wavefront was placed hierarchically upstream of the clock in the CW^L^ model (same hierarchy as in Meinhardt’s RD model). Despite the conceptual similarity between CW^L^ and RD models, Pourquie and colleagues disfavored the RD model. This is because the waves of *c-hairy1* transcription were not disturbed when half of the PSM is removed, showing the waves are kinematic but not propagatory (i.e., requiring information transfer from cell to cell) (*25*). In contrast, the RD model cannot easily explain those results due to its strong dependence on diffusible oscillatory molecules which should be affected by tissue dissections. Furthermore, later studies showed that the segmentation clock oscillates in single dissociated cells (*27**–**29*). These results showed that the clock is likely cell autonomous and is not built on the interaction of diffusible oscillatory molecules or gradients.

### Discovery of the fibroblast growth factor signaling gradient

According to the CW^O^ model, the segmentation clock provides temporal information for segmentation, while the wavefront provides positional (spatial) information (*7*). Soon after the discovery of segmentation clock, in 2001, Pourquie and Takeda labs found that fibroblast growth factor (FGF) signaling establishes a posteroanterior gradient in the PSM in chicken and zebrafish embryos, respectively (Fig. 2A) (*30*, *31*). Activation of FGF signaling expanded the gradient anteriorly and resulted in smaller somites whereas FGF inhibition caused the opposite outcome. These landmark results were taken as evidence for FGF signaling playing a role equivalent to the wavefront for the CW^O^ model. Therefore, a threshold concentration of FGF signaling was proposed to act as the wavefront (i.e., the determination front) in the CW^L^ model. The FGF gradient, and hence the determination front, regresses posteriorly because of tail elongation. At investigated stages, chicken and zebrafish PSM tissues contained four to five predetermined compartments in the aPSM, whose segment sizes cannot be changed by perturbing the FGF signaling. Hence, the determination front is located approximately four to five somite lengths posterior to the last formed somite boundary (Fig. 2A). The position of the determination front approximately corresponds to the zone of sensitivity to heat shock in chicken and zebrafish (*12*, *14*, *30*, *31*).

According to the CW^L^ model, the posteriorly regressing determination front provides cell competence to segment. Once competent, a group of cells can respond to a critical phase of the clock oscillations and commit into segmentation (*1*). Consequently, somite sizes are defined by the distance over which the determination front regresses in a clock cycle. Somite sizes can be changed by changing either the period of the clock or the regression speed of the front.

The CW^L^ model fundamentally differs from the PS model because of the presence of a morphogen gradient encoding positional information whereas that role was attributed to a critical phase match between two clocks in the PS model. Despite conceptual similarities, the CW^L^ model also differs from the CW^O^ model in several key aspects: First, kinematic clock waves were not predicted in the CW^O^ model (Fig. 1A). However, in our opinion, the necessity of kinematic waves for segmentation (as in the PS model) has not been demonstrated (see discussions later in this review). Second, the wavefront was proposed to be an anteroposterior timing gradient in the CW^O^ model (Fig. 1A) (*26*) and Cooke explicitly disfavored a morphogen signaling gradient (*32*). In contrast, the wavefront was proposed to be a posteroanterior signaling gradient in the CW^L^ model (Fig. 2A). Third, the clock and wavefront are independently and serially acting in the CW^O^ model, while the wavefront was proposed to act hierarchically upstream of the clock in the CW^L^ (*25*). Fourth, the CW^O^ model stated the clock gated the spatial information given by a smoothly regressing wavefront (*7*); in contrast, the CW^L^ model stated the wavefront gates the periodic information provided by the clock into space (*1*, *30*).

### Discovery of the Wnt and retinoic acid signaling gradients

After the discovery of FGF signaling gradient, Herrmann and colleagues found that Wnt signaling also establishes a posteroanterior gradient along the PSM, and perturbation of Wnt signaling causes similar effects on somite size to those reported by perturbation of FGF signaling, although the change was local and was over a shorter spatial range (*33*). They also showed that Wnt signaling enhances FGF signaling. In parallel, Storey and colleagues showed that retinoic acid (RA) signaling (which is produced in the somites by Raldh2 enzyme) and FGF signaling antagonize each other’s activity. Correspondingly, smaller somites form in vitamin A–deficient quail embryos, likely due to increased FGF signaling in the PSM (*34*). These results led to the development of a model, in which the determination front is positioned by the activities of three opposing gradients (CW^OG^, “OG” for opposing gradients) (Fig. 2B).

Later studies showed that FGF and Wnt signaling cross activate each other: Genetic disruption of one reduces the other (*33*, *35**–**37*). Also, these perturbations caused axis truncation and segmentation arrest. Thus, activities of these three signaling pathways are entangled in the PSM. This entanglement created a major obstacle in understanding how the positional information is instructed. It was unclear whether the positions of somite boundaries are instructed by one gradient or by a combinatorial effect. Likewise, it was unclear whether the gradient(s) provide positional information at concentration thresholds as in the CW^L^ and CW^OG^ models or by another mechanism. To decipher the mechanism governing somite segmentation, one has to combine time-controlled perturbation experiments with quantitative measurements and computational modeling (*38*).

### POSITIONAL INFORMATION

In this section, we will discuss the mechanism instructing the positional information of somite boundaries and the coinciding molecular discoveries. We begin by discussing where the somite boundaries are determined, followed by a discussion of FGF signaling directly encoding the boundaries. We end with a discussion on how FGF signaling encodes positional information.

#### The position of segment boundaries is instructed in advance

The CW^L^ model stated that the wavefront controlled both the arrest of clock oscillations and determination of segment boundary position (*25*, *31*, *39*). While this statement could be true, in our view, it created unintended confusion in the field. Consequently, rostrocaudally (RC) polarized striped expression of clock (*Hes/her*) and/or *mesp* genes in the aPSM was accepted as a proxy for segmental determination (*39*). This is misleading because the arrest of oscillations and expression of *mesp* genes occur in the aPSM (*40**–**42*), whereas the boundary commitment happens in the mid-PSM of chicken (*30*), zebrafish (*31*, *43*, *44*). In *Fgfr1* mutant mice, FGF targets are affected at around 5-somite stage, whereas somite disruption begins at 10-somite stage (*36*). Likewise, inhibition of FGF signaling could not notably change the size of the first three prospective somites in in vitro human embryo models (*45*). These data suggest that the determination front likely lies posterior to the expression domain of *Mesp* genes in mice and human models as well. However, more quantitative studies are required to determine where the segmental determination occurs throughout the somitogenesis in mice and human embryo models. We argue that without looking at the right markers at the right positions, one cannot mechanistically understand how somite segmentation is instructed (*46*).

In the aPSM, the segmentation clock and Mesp proteins (along with other regulators) establish RC polarity of somites (*47**–**51*), which is important for differentiation of segmented cells. Mesp proteins are also necessary for formation of segment boundaries in mice (*42*) and likely regulate segment boundary formation redundantly with an unknown mechanism in zebrafish (*49*). Thus, Mesp proteins execute the segmental commitment decision that is instructed earlier by the segmentation clock and signaling gradient(s).

### FGF signaling encodes positional information for segment boundaries

Later studies showed that inhibition of RA signaling in whole embryos and surgical removal of RA-synthesizing cells (somites) in three-dimensional PSM explants did not change segment sizes in zebrafish. These results showed that RA signaling is dispensable for instructing the position of determination front in zebrafish (*44*). Likewise, RA signaling is needed for body extension only during early stages in mice (*52*, *53*), and several somites form in *Raldh2* mutants in mice (*54*). Similarly, vitamin A–deficient quails still form somites, albeit with 10% reduction in sizes (*34*). Similarly, supplementing in vitro human embryo models with RA did not change somite sizes (*55*). These results suggest that, although RA signaling might fine-tune the positions of some boundaries in amniotes, it acts indirectly, likely through FGF signaling (*56*). Primary instruction for the determination front is not delivered by the RA gradient.

By using inducible transgenic animals, the activities of FGF and Wnt signaling were abruptly blocked during mid-somitogenesis stages in zebrafish. Blocking each one of the signaling pathways rapidly decreased the activity of the other, highlighting the strong cross-talk between the two. In contrast, somite boundary changes occurred much later upon inhibition of Wnt signaling compared to that of FGF signaling (*44*, *57*). These results showed that, while FGF signaling and the segmentation clock instructs segment boundaries at a similar position (*31*, *44*, *47*, *58*), Wnt signaling acts at a more posterior position than the determination front. This suggests that FGF signaling directly encodes positional information for segment boundaries, while Wnt signaling indirectly and permissively affects segment boundaries through FGF signaling in zebrafish (*44*). Recent results obtained in human embryo models confirmed these earlier conclusions from zebrafish: Inhibition of FGF signaling changed somite sizes only after three predetermined somites form, but inhibition of WNT signaling could not change somite sizes during the course of these experiments (*45*).

### The determination front is non-cell autonomously instructed

**Fig. 3.**
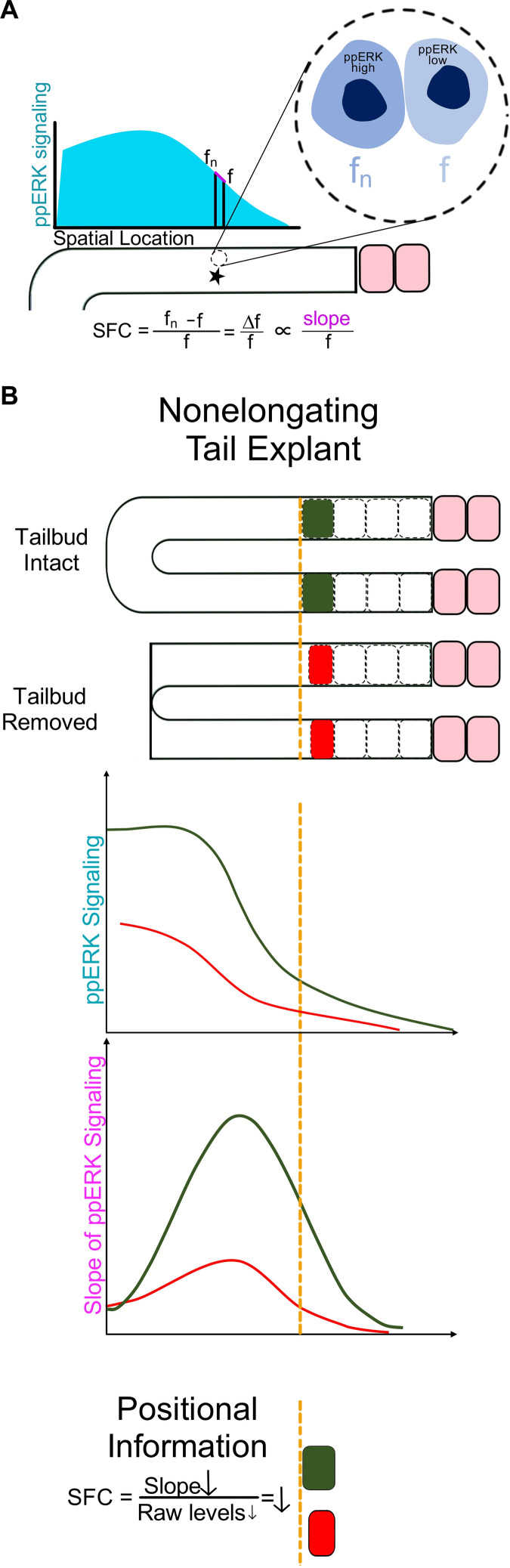
SFC mechanism for boundary determination uniquely explains an experiment in nonelongating PSM explants. (**A**) SFC detection of ppERK gradient (light blue) hypothesizes neighbor-to-neighbor comparison of signal levels (*f*_n_ versus *f*) for determination front (black star). Ratiometric comparison of fold change is mathematically equivalent to measuring gradient slope (magenta) over raw signal (*f*) ratio. *Y* axis represents ppERK levels; *x* axis represents space in the PSM. (**B**) Removal of the tailbud from nonelongating explant (red lines) decreased both the ppERK level and its slope at the determination front compared to intact nonelongating explant (green lines). Despite reduced ppERK and contrary to the prediction of the CW model, tailbud-less explants made smaller somites. The outcome supports the SFC mechanism: The slope decreased more drastically than the ppERK levels; their ratio decreased, shifted the critical SFC position, and resulted in smaller somites (red box as compared to green box). Orange dashed line refers to the position of the next predetermined boundary in control intact explants.

The famous French Flag model posits that positional information is provided at threshold concentrations of morphogen gradients. This idea is adapted for somite segmentation by stating that cells are instructed to form segments at a position where the wavefront activity falls below a threshold level (CW^L^ model) (*30*, *31*). We therefore tested whether the major readout of the FGF signaling in the PSM [activated double-phosphrylated extracellular signal–regulated kinase (ERK), i.e., ppERK] matches to a fixed threshold level at the determination front position throughout zebrafish somitogenesis. We found that the ppERK levels at the determination front changed markedly among stages. The slope of the ppERK gradient also changed in a similar fashion. The ratio of the slope to concentration level (named the spatial fold change or SFC) remained, however, fixed at the determination front position throughout somitogenesis (*44*). The SFC mechanism is equivalent to a ratiometric detection of ppERK among neighboring cells (Fig. 3A).

We then performed several surgical, pharmacological, inducible genetic, and mosaic perturbation experiments. Different perturbations, all decreasing ppERK levels, affected somites sizes differentially: Some increased, some decreased, and some did not change somite sizes (*59*). However, in all experiments, when the critical SFC shifted to different locations in the PSM, the positions of segment boundaries followed the SFC shift (*44*). For example, a decrease in ppERK activity usually increases somite lengths (*30*, *31*). Therefore, formation of smaller somites in tailbud-less PSM explants is contradictory with the concentration threshold prediction of the CW^L^ model (Fig. 3B). Measurements of ppERK in tailbud-less explants showed the slope of the gradient decreased more markedly than the ppERK levels; therefore, the critical SFC position shifted less posteriorly in comparison to control ones, and consequently, somite sizes decreased in tailbud-less explants (Fig. 3B) (*44*). Collectively, results of several perturbation experiments showed that the FGF/ERK pathway does not encode positional information cell autonomously at a threshold concentration as posited by the CW^L^ and CW^OG^ models (*30*, *31*, *33*). Instead, segment boundaries are non-cell autonomously instructed by a threshold ratio of ERK activity among neighboring cells (*44*).

Currently, we still do not know how the positional information encoded by the SFC of ppERK is decoded by the cells. A marker for the determination front has so far not been found (see the “Future directions” section).

### TEMPORAL INFORMATION

This section will discuss the mechanisms that encode the temporal information of somitogenesis. To do so effectively, we will discuss conditions needed for the generation of oscillations. This will lead to a discussion on the potential pacemaker of the segmentation clock.

### Genes displaying oscillatory transcription differ among vertebrates

After the discovery of the first segmentation clock gene, studies identified many genes with oscillatory expression in the PSM of zebrafish, chicken and mice embryos, as well as in human embryo models (*1*). Overall, these studies showed that the number and identity of oscillating genes varies among vertebrates. The number of oscillating genes seems to increase with the period of somite segmentation in each species [zebrafish has the lowest number, followed by chick, mice (*60*), and human embryo models (*61*)]. We hypothesize that these differences might partially result from two factors: First, the segmentation clock might regulate more genes transcriptionally in some species. Second, RNA and protein stabilities differ among vertebrates (*58*, *62**–**64*). Even if orthologs of a gene are transcriptionally regulated by the segmentation clock across the species, transcripts of that target gene might not oscillate in a species with a faster ticking clock because of the difficulty of proportionally increasing all biochemical reaction rates (see next section). Comparative data among mammalian species shows that biochemical reactions rates of some, but not all, genes scale with the segmentation clock period between mice and human (*64*). We also hypothesize that the number of oscillating genes will eventually saturate to its highest value beyond a critical clock period.

To fully comprehend an oscillatory process, say somitogenesis, there is a need to correctly identify its pacemaker (see next section). This will require assessment of the functional role and autonomy of key oscillating genes in the system (*65*).

### Conditions to generate oscillations and multiple categories of oscillatory genes

As Lewis summarized (*66*), generation of oscillations requires many conditions to be satisfied (*66*, *67*): (i) a negative feedback loop, (ii) time delays in the production of molecules, (iii) nonlinearity in the system, (iv) strong production rates, and (v) short-lived molecules. For example, if an autoinhibitory transcriptional feedback loop exists in an oscillatory system, then transcription will be initiated when short-lived repressors are degraded. If the transcription rate is strong, and the time delay between the initiation of transcription and synthesis of sufficient repressor proteins is long enough, then the RNA levels will rapidly increase. Then, the RNA levels overshoot a steady-state intermediate level that would otherwise be observed in the absence of a negative feedback loop (*68*). Later, high RNA levels will result in high repressor proteins. Repressors will shut off their own transcription. Because of short half-lives, first RNA levels and then protein levels will rapidly decrease. As this cycle continues, it generates sustained oscillatory gene expression, which is also called a limit cycle.

**Fig. 4.**
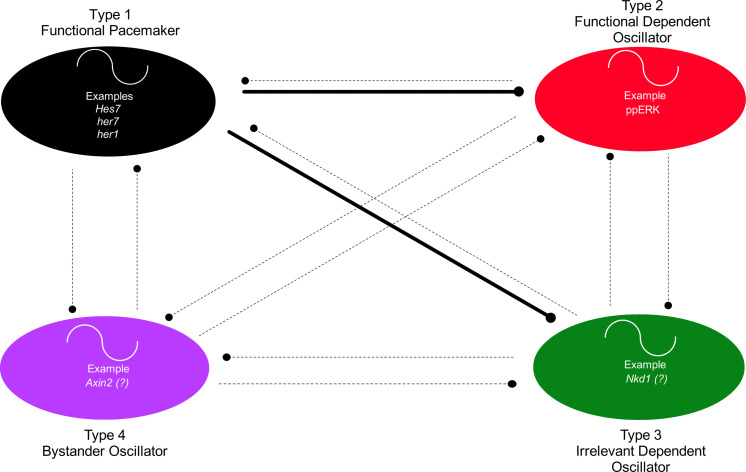
Different types of oscillators. All types of oscillators might be cross-talking with each other (dashed and solid lines). The dots at the end of each line represent regulation of some kind (be it activation or inhibition). Some of the regulations might be important for their oscillations (solid lines), while other regulations might be unimportant (dashed lines). The functional pacemaker does not need to regulate all other oscillators. Oscillations of only type I and type II oscillators are relevant for the system function.

In a biological process governed by an oscillatory system, there can be several types of oscillating genes (Fig. 4). Here, we group them into four types: Type 1 oscillator is the major pacemaker that establishes its own oscillations by satisfying the conditions discussed above. Oscillatory expression of the pacemaker is crucial and functionally necessary for the dynamic phenotype of the system. Types 2 and 3 are dependent oscillators that completely rely on their regulation by the pacemaker. However, oscillatory dynamics of only type 2, but not type 3, are functionally necessary for the system phenotype. Type 4 are bystander oscillators whose oscillations are not driven by the major pacemaker but their oscillatory expression are also not necessary for the system phenotype. Also, some of the bystander oscillators might set the pace of oscillations of other bystander ones (i.e., there could be a hierarchy among them). Therefore, the major pacemaker in a system does not need to drive all oscillators.

### The pacemaker of the segmentation clock

Oscillations of only Hes/Her family transcription factors and Delta ligands are conserved among studied vertebrates (*1*, *39*, *60*, *69*). Their mutations consistently result in segmentation defects (*68*, *70**–**73*). *Hes/her* genes inhibit their own transcription and thereby establish the necessary feedback loop as shown in both zebrafish and mice (*65*, *74*, *75*). RNAs and proteins of *Hes/her* genes are also short lived in both zebrafish and mice (*58*, *62*, *63*). Stabilization of Hes7 dampens the oscillations and causes segmentation defects in mice (*76*, *77*). Hes/Her transcription factors act as dimers and satisfies the necessary nonlinearity condition (*77**–**80*). Modifying dimer types and levels changes the period of segmentation in zebrafish (*81*). There are adequate time delays in the feedback loop (*80*, *82*). Changing the time delays in the Hes7 feedback loop changes the period of oscillations in mice (*83*, *84*). Strong transcription rates are shown to be necessary for their oscillations in zebrafish (*68*, *85*). Hes/her transcription factors act cell autonomously; Kageyama, Oates, and Pourquie labs showed oscillatory expression of *Hes/her* genes in single cells testifying to the cell autonomy of the pacemaker (*27**–**29*, *86*). Overall, the *Hes/her* genes satisfy all the conditions to be the pacemaker of the segmentation clock (*66*, *87*, *88*).

In amniotes, in addition to Hes/Delta oscillations, several targets of FGF and Wnt signaling also oscillate (*33*, *60*, *89*, *90*). Kageyama and colleagues showed that ppERK activity oscillates under the control of Hes7 in mice (*21*). Recently, it was also shown that ppERK also oscillates under the control of Her1/Her7 in zebrafish (*46*) and that periodic inhibition of ppERK by Her1/Her7 is functionally important (*46*). These results suggest that ppERK is a type 2 oscillator whose dynamics is critical for somite segmentation. Both the cross-talk between FGF and Wnt signaling and the dependence of ppERK oscillator on Hes7 can explain why oscillations of some Wnt signaling targets (e.g., *Nkd1*) are also controlled by the Hes7 oscillator in mice (*91*). Because their oscillations have, so far, not been shown to be important for somite segmentation (*92*), we hypothesize that they are type 3 oscillators.

One of the objections raised against the Hes/Her oscillator being the conserved pacemaker of somitogenesis in all vertebrates is that transcription of *Axin2* still oscillates in *Hes7* mutant mice (*76*, *93*). The mechanism driving Hes7-independent Wnt oscillators is currently unknown. Nuclear β-Catenin, Wnt signaling effector molecule, levels do not oscillate in zebrafish (*46*). Similarly, its oscillation has so far not been observed in mice (*35*). However, this could be due to inadequate resolution of data and sample sizes. Alternatively, a posttranslationally modified variant of β-Catenin might be oscillating and might be missed with previous quantifications. Therefore, it is currently unknown whether oscillations of Wnt signaling targets depend on an autonomous β-Catenin–dependent negative feedback loop in mice. Notably, oscillation of *Axin2* is lost in *Psen1/Psen2* mutants in mice (*94*), suggesting that severe loss of Notch signaling (and potentially expression of *Hes* family genes altogether) might eliminate all cyclic activity in mice. In addition to *Hes7*, expression of *Hes1* and *Hes5* also oscillate in wild-type mice. It is possible that oscillations of all Wnt signaling targets might be lost in compound mutants of all three *Hes* genes in mice. Alternatively, *Psen1/Psen2* mutants might reflect a Hes-independent contribution of Notch signaling or a Notch-independent contribution of Presenilin in *Axin2* expression. Thus, it remains to be determined whether *Axin2* oscillations are autonomous or dependent on Notch signaling.

Aulehla and colleagues investigated the relevance of Wnt oscillators for somitogenesis. They performed pulsatile pharmaceutical perturbations of Wnt and Notch signaling activities that disrupted coexpression of Lfng and Axin2 in aPSM and resulted in segmentation defects in an in vitro monolayer system (*22*). However, this experiment jointly perturbed two pathways (not just Wnt signaling), and it did not demonstrate whether Wnt oscillations throughout the PSM are needed for somitogenesis. This phenotype can also be explained by an alternative hypothesis: Combined local action of Notch and Wnt signaling in the middle PSM (through affecting ppERK) or in the aPSM (through affecting other molecules) might be needed for somitogenesis. In contrast to this work, perturbation of Wnt signaling alone (both genetically or pharmaceutically) did not prevent somite segmentation both in zebrafish (*44*, *57*) and in vitro human embryo models (*45*). Furthermore, loss of expression of several oscillatory Wnt targets (*Axin2*, *Dact1*, *Dkk1*, and *Sp5*) does not specifically disrupt somite segmentation in mice (*93*, *95**–**98*). Although, this outcome could be attributed to hidden redundancy in the network, genetic evidence to show that Wnt signaling oscillations are necessary for somite segmentation in any species is missing so far. Thus, more studies are needed to reveal both the mechanism driving some of the Wnt oscillators and the relevance of their oscillatory expression for somite segmentation.

A second objection raised against the Hes/Her oscillator being the pacemaker of somitogenesis is presence of partial segment boundaries and irregular clumps of epithelial cells in *Hes7* (in mice) and *her1;her7* (in zebrafish) mutants (*93*). Several arguments can be given against this objection: (i) Epithelization is a separate process from segmentation as seen in *Paraxis* mutants in mice (*99*). (ii) Cells turn on genes involved in epithelization in the aPSM after FGF/Wnt gradients diminish. Therefore, it is expected that these uninstructed cells will be aggregated into irregular clumps in *Hes/her* mutants. (iii) Formation of irregular clumps are also observed at an ectopic location in chicken embryos when Bmp signaling was inhibited (*100*). As argued previously, those clumps do not represent sequential segmentation of somites (*93*). (iv) This phenotype is not unique to *Hes/her* mutants. Irregular clumps of epithelial cells are also detected in *Fgf4;Fgf8* mutants (*37*) and conditional gain and loss of function of β-Catenin mutants in mice (*101*). Therefore, disruptions of both the clock and the gradients result in the same phenotype. Together, these results suggest that the clock and the gradient control sequential segmentation of regular-sized somites. In their absence, cells still clump as long as they express genes driving epithelization. This often results in pebble-beach (i.e., clump-like) scoliosis phenotypes described in patients (*102*).

Pourquie and Aulehla labs have been investigating the role of metabolism in somite segmentation. They have found that metabolic gradients are established in the PSM (*103*, *104*) and they are linked to FGF and Wnt signaling gradients (*104*, *105*). Inhibiting respiration disturbed segmentation clock oscillations and somite segmentation (*104*). Therefore, one scenario is that a so far hidden metabolic clock might be pacing oscillations of all oscillating targets. Future research might test the validity of this scenario. Science progresses by repeated attempts to falsify existing models. If *Hes/Her* oscillator is claimed not to be the conserved pacemaker, then alternative molecular models should be proposed so that they can be tested side by side in future studies.

### Notch signaling synchronizes oscillations of the segmentation clock among neighboring cells

Delta/Notch signaling is critical for somite segmentation; mutations of several genes involved in the pathway are linked to segmentation defects both in animal models and patients with congenital scoliosis (*1*, *106*). Oscillations of Delta expression and Notch activity are driven by the cell autonomous Hes/Her oscillators in zebrafish and mice (*58*, *107*). Expression of *Hes/her* genes oscillating in single cells suggests that oscillation of Notch activity is not necessary for cell autonomous Hes/Her oscillations. As Lewis and colleagues hypothesized, Delta/Notch signaling couples the Hes/Her oscillators among close neighboring cells and thereby locally synchronizes clock oscillations in the PSM (*108*). Disruption of Delta/Notch oscillations, either in its loss-of-function mutants or in constitutively overexpressing transgenic embryos, gradually desynchronizes oscillations among the PSM cells and results in segmentation defects after formation of several normal somites (*50*, *109**–**112*). By the nature of coupling nearby oscillators, Delta/Notch signaling can also modify the collective period of oscillations (*113**–**118*).

It has been debated whether the role of Notch signaling differs among species (*1*, *106*). As discussed in the preceding sessions, establishment of oscillators requires strong production rates. Segmentation usually proceeds normally in heterozygous *her1/her7* mutants in zebrafish, but disruption of the cofiring of clock genes in certain mutants results in segmentation defects (*68*, *85*). Similarly, Delta/Notch signaling activates transcription of *Hes/her* genes, but the contribution of this activation might slightly differ among vertebrates. If Notch signaling is severely disrupted in some genetic or pharmacological perturbations, the strength of *Hes/her* transcription might decrease below a critical level to sustain limit cycle oscillations (*28*). In mice, *Psen1/Psen2* mutants seems to satisfy this condition (*94*), though a Notch-independent role of Presenilin has so far not been ruled out (because of the presence of single cell oscillations in vitro). In zebrafish, transcription rates of *her1/her7* do not fall adequately to arrest their oscillations in Delta/Notch mutants or when Notch signaling is pharmacologically inhibited (*111*, *112*, *119*).

### RECENT MODELS TO EXPLAIN INTEGRATION OF POSITIONAL AND TEMPORAL INFORMATION

Feynman once famously wrote on his blackboard “What I cannot create, I do not understand” (*120*). To achieve a mechanistic understanding of somitogenesis, one needs to reengineer somite segmentation in vivo by using necessary molecular ingredients and according to the unique prediction of one model. In this section, we will discuss more recent models proposed to explain somitogenesis. These models build upon earlier experimental discoveries and therefore are more molecular based (in contrast to earlier phenomenological models). We start with a discussion on the progressive oscillatory reaction-diffusion (PORD) model followed by a discussion on a modern adaptation of the PS model. We end with discussing the recently proposed clock and oscillatory gradient model.

### The PORD model

To explain integration of spatiotemporal information, Sharpe and colleagues proposed a PORD model (*121*). In this model, a cell-autonomous activator and a diffusing repressor interact at short range. The repressor is locally produced by the activator stripe located in the aPSM. The levels of the activator are also enhanced by FGF signaling like in Meinhardt’s RD model. In contrast to the RD model, the gradient of FGF signaling is not needed for somitogenesis, but it only helps explaining size regulation in smaller animals. They showed that continuous pharmaceutical inhibition of FGF signaling causes formation of multiple large somites. This outcome can be explained by the PORD model but not by the CW^L^ model. They also showed that splitting the chick PSM into anterior and posterior halves induces ERK activity at the wound boundaries; they suggested that this wound response could explain why the clock waves were not affected by surgical manipulations (*25*). Therefore, they argued that the clock waves are not kinematic but rather propagatory (*121*).

In our opinion, several pieces of evidence disfavor the PORD model: (i) We reproduced formation of multiple large somites upon continuous inhibition of FGF signaling in zebrafish. By quantifying ERK activity (not done in the PORD study), we showed while these results are incompatible with the CW^L^ model, they are explicable with the SFC detection mechanism (*44*). Therefore, explaining these results does not require a propagatory wave model with a yet-to-be found diffusible inhibitor. (ii) When embryos split into two halves, segmentation of the posterior half waits until the anterior half fully segmented both in chicken and zebrafish (*44*, *122*). These experiments cannot easily be explained by the PORD model as it requires fine-tuning of ppERK induction at the wound boundary (to guarantee cut-PSM behaves exactly like an intact PSM). (iii) Clock oscillations are cell autonomous (*27**–**29*, *86*). Sharpe and colleagues modeled this by setting the diffusion rate to zero in their models (*121*). However, if the inhibitor is a secreted diffusible molecule, then it should diffuse away from cultured single cells as well. Therefore, we think cell autonomy of oscillations cannot be explained with the PORD model. (iv) The PORD model claims segmentation would occur with a flat signaling gradient. Although indirect, existing data argue against this: Gain of function of Wnt (and thereby FGF) signaling prevents segmentation (*35*). (v) Like the RD and CW^L^ models, the PORD model claims that FGF signaling acts hierarchically upstream of the clock for instructing somite boundaries. Conversely, a recent study showed that the clock acts upstream of the ERK activity gradient to instruct somite segmentation (*46*).

### Revisiting the PS model

Kageyama and colleagues made the important discovery that both the peak levels and the spatial range of ppERK gradient oscillate in mice PSM (*21*). An oscillatory gradient is hard to reconcile with the CW^L^ model in which a static gradient instructs somite boundaries at a threshold concentration. Nonetheless, they still interpreted this major discovery within the CW^L^ model by switching the spatial and temporal roles of the clock and wavefront. They posited that stripy expression of the Hes/Notch clock provides the spatial information and the spatial-range oscillation of ppERK provides the temporal information (*21*). Accordingly, a stripe of cells in the mid-PSM becomes released from ppERK inhibition and receive Notch activation and thereby are instructed to make a future somite (*123*). Although Niwa *et al.* (*21*) did not refer to the PS model (*20*), Pourquie and colleagues interpreted their description closer to the PS model than the CW^L^ model (*93*) (Fig. 2C). Overall, the hybrid model of Niwa *et al.* (*21*) proposes that the PS of Hes/Notch and ppERK oscillators along the PSM determines segment sizes and timing.

Aulehla and colleagues quantified live oscillations of a Notch signaling target (Lfng) and a Wnt signaling target (Axin2) and showed that these two reporters oscillate out of phase in the pPSM but in phase (i.e., coexpressed in the same cells) in aPSM. They proposed that this half a cycle phase difference is critical for segmentation. Supportively, they performed pulsatile pharmaceutical perturbations of Wnt and Notch signaling which disrupted coexpression of Lfng and Axin2 in aPSM and resulted in segmentation defects in an in vitro monolayer culture system (*22*). This work explicitly cited the PS model (*22*).

However, in our view, several pieces of evidence still argue against the PS model as a foundational model: (i) Experimental support for the PS model comes from in vitro data. In contrast to this, in vivo mesoderm cells at the onset of visible clock oscillations in mice embryos oscillate in synchrony, and the spatial phase difference gradually accumulates to its final level only at the sixth oscillation cycle (*124*). Clearly, mice embryos do not use a particular phase difference to instruct boundaries of the anterior somites at the very least. (ii) Disruption of segmentation could still be explained by alternative mechanisms. It is possible that the phenotype arises because of preventing local coexpression of Notch and Wnt signaling targets instead of changing their PS throughout the PSM. For example, Notch and Wnt signaling might regulate ppERK activity at the determination front in the mid-PSM or RC polarized expression of several genes in the aPSM in mice (*35*, *101*). Supportively, RC polarity was disrupted with simultaneous pulses of drugs inhibiting Notch and activating Wnt signaling (*22*). This phenotype also partially resembles constitutively activated β-Catenin transgenics in which segmentation also fails while kinematic clock waves and their PS occur in the PSM (*35*). Therefore, several processes involved in physical segmentation of somites (alongside the RC polarity) might be disrupted in aPSM after segmental commitment. Ideally, one should not disrupt but instead reconstruct segmentation according to the predictions of the PS model. (iii) As discussed above, oscillatory expression of Wnt targets have so far not been shown to be essential for somitogenesis in mice. Direct support for the Notch-Wnt PS model awaits demonstration of importance of Wnt oscillators for somitogenesis. (iv) So far, no Wnt signaling target is found to be oscillating in zebrafish (*60*). Therefore, this Notch-Wnt PS model, even if it is relevant for amniotes, does currently not provide a fundamental mechanism conserved among vertebrates. Although the in vitro phenotype is exciting, more research is needed to explain this phenotype and to test the possible role of PS of multiple oscillators across the PSM in amniote embryos.

### The clock-dependent oscillatory gradient model

We recently investigated the dynamics of ppERK gradient and the segmentation clock simultaneously in zebrafish embryos. Experiments showed that ppERK oscillates under the inhibitory control of the segmentation in clock wild-type embryos but lost its oscillations in *her1;her7* clock mutants. Thus, the oscillatory dynamics of ppERK and its dependence on Hes/Her clock oscillations are conserved between mice and zebrafish (*46*). Live imaging in zebrafish further showed that the positional information (SFC of ppERK) stalls at a location for approximately half a clock cycle and then jumps to its next posterior location. Later, segment boundaries are formed at these locations (*46*). We then aimed to reengineer somite segmentation in clock mutants by mimicking clock’s impact on the ppERK gradient. We periodically bathed mutant embryos in drugs reducing ERK activity. Experiments successfully reengineered (i) ppERK oscillations, (ii) discrete SFC jumps, and (iii) missing segmentation in mutant embryos (*46*).

**Fig. 5.**
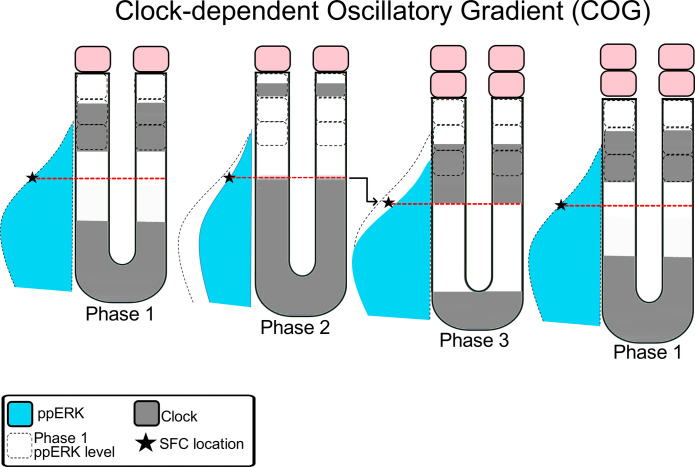
The COG model. Segmentation clock oscillations display kinematic waves (dark gray). The clock inhibits ppERK (blue), resulting in its oscillations. ppERK provides positional information at a critical SFC (star sign). The positional information stands still for part of the clock cycle (between first and second phases) and jumps to a more posterior region (at the third phase). Thereby positional information is discretized by the periodic action of the clock. The solid pink boxes are formed somites. The cartoon represents mid-somitogenesis stage zebrafish embryos containing three predetermined compartments (dashed boxes).

These observations are quite difficult to be reconciled by the existing models. For instance, experiments showed the clock hierarchically lies upstream of the ppERK gradient, in contrast to the RD, CW^L^, and PORD models. Furthermore, an oscillatory gradient cannot encode positional information at a concentration threshold as posited by the CW^L^ model, because that threshold will fluctuate back and forth in the PSM because of oscillatory activity and tail elongation (*46*). Lastly, because somite segmentation is reengineered by globally inhibiting ppERK, in the absence of any molecular clock and kinematic waves, this observation is very difficult to be explained by the PS and RD models (in which kinematic waves are essential). Together these recent findings necessitated the need to propose a new model called the clock-dependent oscillatory gradient (COG) (*46*). According to the COG model, the major role of the clock is to drive oscillatory activity of the ppERK gradient and thereby periodically discretize positional information (Fig. 5).

The COG model currently builds upon experiments performed in zebrafish, yet with molecular dynamics conserved between zebrafish and mice. Future experiments will assess whether reengineering somitogenesis is possible according to any of the other proposed models in multiple species. In addition, future experiments will assess whether and how the COG model can explain periodic occurrence of heat shock–induced segmentation defects in wild-type embryos (see the “Future directions” section).

### FUTURE DIRECTIONS

#### The decoding and execution of spatiotemporal information

**Fig. 6.**
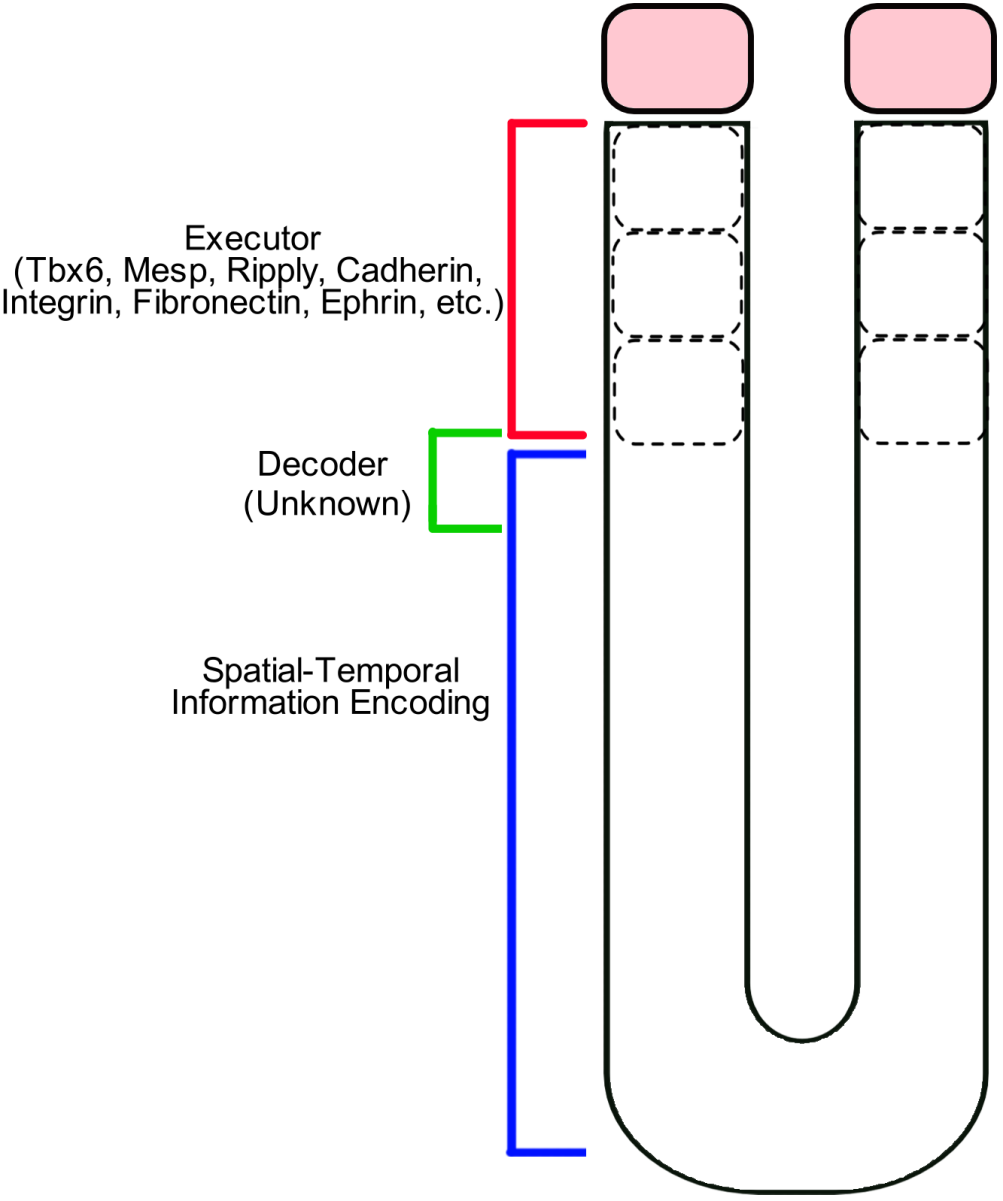
Modular division of steps for somite segmentation. The spatiotemporal control of somite segmentation can be divided into three different modular steps: (i) information encoding by the clock and oscillatory gradient, (ii) decoding this information, and (iii) executing the segmentation decision.

Somitogenesis involves multiple distinct steps (Fig. 6). The spatiotemporal information, encoded by the segmentation clock and the ERK activity gradient, are integrated to instruct segment boundaries in the zebrafish mid-PSM. Currently, it is not known how cells decode this spatiotemporal information. Mosaic experiments suggest cells determine segment boundaries non-cell autonomously (*44*). Therefore, the signal decoder machinery likely uses cell membrane proteins. Several membrane proteins, including ephrins, cadherins, and integrins, might play a role in the decoding process. Ongoing studies will explain the molecular mechanism decoding this segmental commitment in the mid-PSM and executing somite segmentation and RC polarization in the aPSM (Fig. 6).

*Tbx6* is a critical transcription factor expressed in the PSM (*125*, *126*). Mutation of *Tbx6* results in defects in both segmentation and RC polarization of somites in both zebrafish (*125*) and mice (*126*, *127*). The anterior border of the Tbx6 protein expression domain coincides with later somite boundaries in both mice and zebrafish (*128*, *129*). An ectopic Tbx6 border can generate a border resembling a somite boundary in zebrafish (*130*). Because the anterior Tbx6 border forms one to two somite lengths anterior to the determination front in zebrafish, in our opinion, the Tbx6 border does not seem to be the hidden decoder sought after. However, the Tbx6 border is likely to be one of the critical executors of boundary formation. Future research will elucidate the missing link between the encoded segmental commitment and the Tbx6 border (Fig. 6).

Another important question that has not been completely resolved is the reconstruction of the gene regulatory network executing somite segmentation in the aPSM. Tbx6, and Mesp- and Ripply- family proteins are critical for somite boundary formation and, directly or indirectly, cross-regulate each other (*47*, *125*, *127**–**132*). Although several biological cartoon and mathematical models have been developed to explain somite boundary formation and establishment of RC polarity, we view that these important questions have not been resolved yet.

### Experimental observations still awaiting explanations

The cause of periodic segmentation defects generated by a single pulse of heat shock still awaits a mechanistic explanation (*14*, *133*). While we agree with Stern that these periodic defects cannot be accurately explained by a static wavefront of the CW^O^ and CW^L^ models, they cannot be explained by the CC model either. However, they may be hypothetically explained by the oscillatory ERK gradient observed in both zebrafish and mice. If a strong heat shock disturbs the ERK activity, because of its oscillatory nature, these disturbances could repeat multiple times in some embryos until they are dampened and the ERK activity recovers back to its normal oscillatory activity purely driven by the segmentation clock. Supporting this hypothesis, some repetitive segment size anomalies have been reported when ERK activity was disturbed with drugs briefly (*134*). We hope future investigations will reveal the molecular mechanism underlying heat shock–induced segmentation anomalies.

Segmentation of the first five somites at very close time intervals in chicken embryos also awaits an explanation (*100*). Stern used this data to argue that the first few segments might form independent of a molecular clock. However, anterior somites form sequentially in zebrafish and mice. The segmentation clock is also active before formation of first somites in zebrafish, chicken, and mice (*110*, *124*, *135*). There is no direct evidence to support that first somites form by a clock-independent mechanism in chicken embryos. Furthermore, segmental determination models should attempt to explain the commitment of a group of cells to segmentation at the determination front in mid-PSM. Physical segmentation of somites requires sequential actions of a decoder in mid-PSM and an executer in aPSM (Fig. 6). We hypothesize that the executer mechanism is stalled (or not active yet) in chicken embryos until the first five segments are predetermined, afterwards the executer mechanism jump-starts, and all five somites form almost simultaneously. Supporting this speculation, a similar jump-start mechanism acts for the initiation of muscle differentiation (but not segmentation) in zebrafish: Expression of *myoD* and several other genes initiate simultaneously in the first seven to eight segmented somites (*136*). Therefore, chicken-specific near-simultaneous segmentation of first five somites requires further investigations and cannot be used as an argument against any model (including the CC model). Future research is necessary to understand the mechanism driving rapid segmentation of anterior-most somites in chicken embryos.

### Human somitogenesis

Establishment of in vitro human embryo models allows investigation of early stages of human development that was previously inaccessible to experimental manipulations. Several labs showed that culturing induced pluripotent stem cells under suitable cocktails of media and extracellular environment directs them into the PSM fate. These human embryo models display kinematic waves of clock oscillations and form somite-like segments at their anterior ends (*45*, *48*, *55*, *137*). Recent studies in human embryo models have already started to confirm and extend the knowledge gained from in vivo studies of animal models.

By performing cell tracking experiments, Pourquie lab found that RC polarized expression of Mesp2 is accomplished by cell sorting (*48*). Mesp2-expressing cells are initially intermingled with nonexpressing ones in a large domain but later sort out to form a compartment corresponding to anterior half of the next-forming somite in the aPSM. Future cell tracking studies in animal embryos will assess whether this previously unknown cell-sorting mechanism is conserved in all vertebrates or is specific to some of them. Pourquie lab also showed that inhibition of CC does not abolish segmentation clock oscillations and there is no correlation between CC phases and segmentation clock phases in human embryo models (*16*), agreeing with earlier zebrafish *emi1* mutant results and provides additional evidence against the CC model (*19*). Ramanathan lab investigated the roles of FGF and Wnt signaling in instructing somite sizes as well as in affecting kinematic clock waves (*45*). They found that FGF, but not Wnt, signaling directly controls positions of somite borders in human embryo models. This conclusion confirms earlier results obtained in zebrafish embryos (*44*). They also showed that FGF, but not Wnt, signaling affects the kinematic clock waves, agreeing with an earlier result obtained in mouse tailbud explants (*16*). Alev lab investigated the influence of RA signaling in human somitogenesis (*55*). Perturbation of RA signaling changes somite sizes only mildly (~10%) and only in some species (*34*). They also found that supplementing culture media with RA did not change segment sizes but it improved epithelization of somites (*55*).

in vitro embryo models are also used to test the role that metabolism plays in affecting the timing of somite segmentation across species. Pourquie and colleagues showed that metabolic rates are faster in mouse than human models, scaling with their segmentation clock period (*138*). We still know very little about human somitogenesis; future studies will expand our knowledge and also allow for comparison of the mechanisms controlling somite segmentation across several vertebrates (*139*).

Currently, human embryo models have not generated PSM together with all of flanking tissues (lateral plate mesoderm, neural tube, ectoderm, and notochord). Perhaps, because of absence of neighboring tissues, somite shapes and structure are less regular in human embryo models as compared to those seen in vivo. Likewise, the mechanical forces and cytoskeletal functions might be not fully optimized yet in the human embryo models. These differences could potentially result in epiphenomenon (i.e., exaggerating the roles of different molecular machinery as compared to in vivo embryos). Ongoing efforts in this field will hopefully increase the robustness of in vitro embryo models to the level of in vivo embryos.

## DISCUSSION

### Outstanding questions

**Fig. 7.**
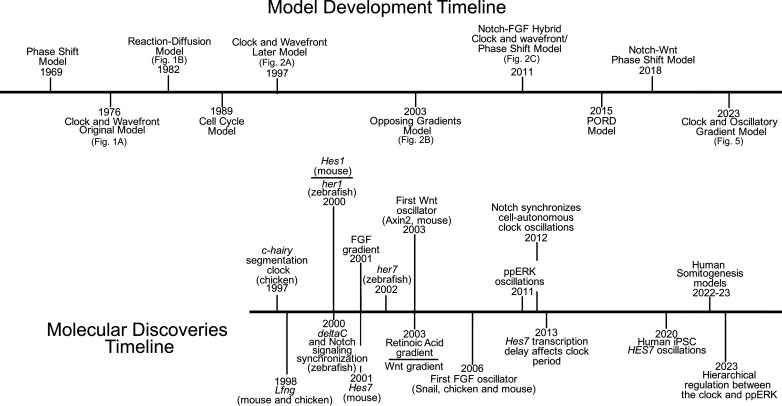
Timeline of notable discoveries and model developments. The timeline of somitogenesis, presenting models (top) and molecular discoveries (bottom). The models refer to preceding figure panels where applicable.

Our understanding of somitogenesis advanced substantially since the very first models proposed more than half a century ago (Fig. 7). Molecular discoveries beginning with the discovery of segmentation clock in 1997 paved the way for this progress. Recent advances in microscopy and quantitative biology enabled mechanistically grasping this mesmerizing patterning process of early vertebrate embryo and creating somites at will with such informed approach (Fig. 7). Nevertheless, several important questions are outstanding in the field: (i) What is the mechanism controlling somite numbers in each species? Can it be drastically changed? (ii) What posttranscriptional and posttranslational mechanisms trigger rapid degradation of oscillatory RNA and proteins (*140*)? (iii) What is the molecular mechanism by which the clock triggers ppERK oscillations (*141*)? (iv) What is the decoding mechanism reading out positional information downstream of ppERK? (v) How is the decoder coupled to the mechanism executing physical segmentation of somites? (vi) What is the gene regulatory network establishing RC polarity of somites in the aPSM? (vii) What is the pacemaker of the segmentation clock? Is there any hidden upstream clock controlling oscillations of the rest? (viii) What is the molecular mechanism generating kinematic clock waves along the PSM? Are the clock waves generated by a cell-autonomous timer mechanism (*86*) or by signaling gradients (*16*, *28*, *45*, *141*)? If it is the former scenario, what is this hidden timer? If it is the latter one, which signaling and what feature of it regulates the waves (*141*)? (ix) What is the role of metabolism and mechanics in axis elongation and somite segmentation (*1*)? We hope that coordinated efforts from multiple labs using several model organisms and human embryo models will answer these questions in the next decade.
